# Myocardial Arterial Spin Labeling with Double Inversion Recovery for reduced physiological noise

**DOI:** 10.1002/mrm.70018

**Published:** 2025-08-11

**Authors:** Maša Božić‐Iven, Stanislas Rapacchi, Yi Zhang, Qian Tao, Lothar Rudi Schad, Sebastian Weingärtner

**Affiliations:** ^1^ Department of Imaging Physics Delft University of Technology Delft The Netherlands; ^2^ Computer Assisted Clinical Medicine, Medical Faculty Mannheim Heidelberg University Mannheim Germany; ^3^ Mannheim Institute for Intelligent Systems in Medicine, Medical Faculty Mannheim Heidelberg University Mannheim Germany; ^4^ Department of Diagnostic and Interventional Radiology Lausanne University Hospital (CHUV) Lausanne Switzerland

**Keywords:** myocardial arterial spin labeling, double inversion recovery, flow‐sensitive alternating inversion recovery, cardiac magnetic resonance imaging, myocardial blood flow

## Abstract

**Purpose:**

To introduce Double Inversion Recovery (DIR) preparations for myocardial Arterial Spin Labeling (myoASL) for mitigation of heart rate (HR) variability induced physiological noise (PN).

**Methods:**

DIR‐labeling was implemented for double ECG‐gated myoASL‐sequences and compared with conventional Flow‐sensitive Alternating Inversion Recovery (FAIR) labeling using single inversions. In DIR‐preparations, the FAIR‐inversion pulses were immediately followed by an identical reinversion pulse, applied either slice‐selectively or nonselectively. Bloch‐equation‐based simulation and phantom experiments were performed to evaluate the PN and SNR across a range of HR variabilities. Data from six healthy subjects were acquired to evaluate myocardial blood flow (MBF), PN, and SNR in vivo.

**Results:**

Simulation experiments showed that the average MBF values remained nearly constant across the range of HR variabilities and were comparable across all three sequences. However, DIR‐labeling allowed for greater recovery of the myocardial background signal, which mitigates the sensitivity to HR‐dependent changes in the inversion time. Consequently, PN in the presence of HR variability was substantially reduced with DIR‐labeling. For HR variabilities corresponding to the mean value observed in vivo, this resulted in a simulated SNR gain of 1.79 ± 0.90 for selective and 1.55 ± 0.77 for nonselective DIR‐labeling. In vivo, DIR‐labeling showed reduced PN, with 53% (p<0.05)/44% (p=0.16) less PN compared with conventional FAIR‐myoASL, leading to an average SNR gain of 1.47 ± 0.63 (p=0.09)/1.32 ± 0.57 (p=0.84) with selective/nonselective reinversions.

**Conclusion:**

The proposed DIR‐preparations reduce sensitivity to HR variations and alleviate PN in double ECG‐gated myoASL, improving the precision of myoASL‐based perfusion quantification.

## 1 INTRODUCTION

Myocardial Arterial Spin Labeling (myoASL) has emerged as a promising alternative to first‐pass perfusion cardiac MR (CMR), the clinical gold standard for myocardial perfusion imaging^1^, ^2^. Instead of exogenous contrast agents, myoASL utilizes magnetically labeled blood as an endogenous tracer to enable quantification of myocardial perfusion.^3^, ^4^ Contrast‐free alternatives to perfusion are timely because of the safety concerns attributed to gadolinium‐based contrast agents (GBCAs). These include the risk of nephrogenic system fibrosis in patients with renal failure^5^, ^6^ and inadvertent gadolinium accumulation in the body,^7^ limiting clinical applicability and repeated use of first‐pass perfusion CMR.

Flow‐sensitive Alternating Inversion Recovery (FAIR) preparations are most commonly used in myoASL due to their overall robustness to motion and independence of the vessel geometry.^8^, ^9^ In FAIR‐labeling, magnetization is inverted following the trigger signal in a first heartbeat, followed by the imaging readout in the next heartbeat. In an alternating fashion, the magnetization inversion is performed either slice‐selectively or nonselectively. A control image is obtained with slice‐selective inversion as noninverted spins flow into the imaging slice. Nonselective inversion produces a tag image with the inflow of inverted spins. The difference between the alternating images generates perfusion‐weighted contrast.^9^

However, the inherently low signal‐to‐noise ratio (SNR) impairs the robustness of myoASL and hampers its widespread clinical translation. In FAIR‐myoASL, the noise profile is primarily driven by physiological rather than thermal noise.^10^ The physiological noise (PN) level of double ECG‐gated FAIR‐myoASL is particularly susceptible to heart rate (HR) variations because both labeling and acquisition are triggered to the same cardiac phase.^11^ Thus, changes in the HR lead to a variable inversion time (TI) and inconsistent inversion recovery of the signal across the FAIR images. While the signal of static myocardial tissue is assumed to cancel out in the subtraction of control and tag images, differences in TI between these acquisitions result in different levels of signal recovery after the inversion. In the subtraction, this mismatch causes a residual signal, which manifests as measurement noise.

We hypothesize that the impact of HR variations, causing TI differences between myoASL image pairs, on perfusion quantification can be mitigated using additional inversion pulses immediately after the FAIR‐labeling. Similar to double‐inversion recovery (DIR) black‐blood preparations in cardiovascular MR (CMR),^12^, ^13^ the magnetization within the static myocardial tissue is restored, leading to effective cancellation after the subtraction. We investigate the potential of this approach to reduce PN originating from variable TI in double ECG‐gated FAIR‐myoASL. Simulation and phantom experiments are performed to evaluate the bias and SNR performance of DIR‐preparations. Finally, we assess the effectiveness of DIR‐labeling in reducing PN and improving SNR relative to conventional FAIR‐myoASL in healthy volunteers.

## 2 METHODS

### 2.1 Double ECG‐gated FAIR‐myoASL

**FIGURE 1:**
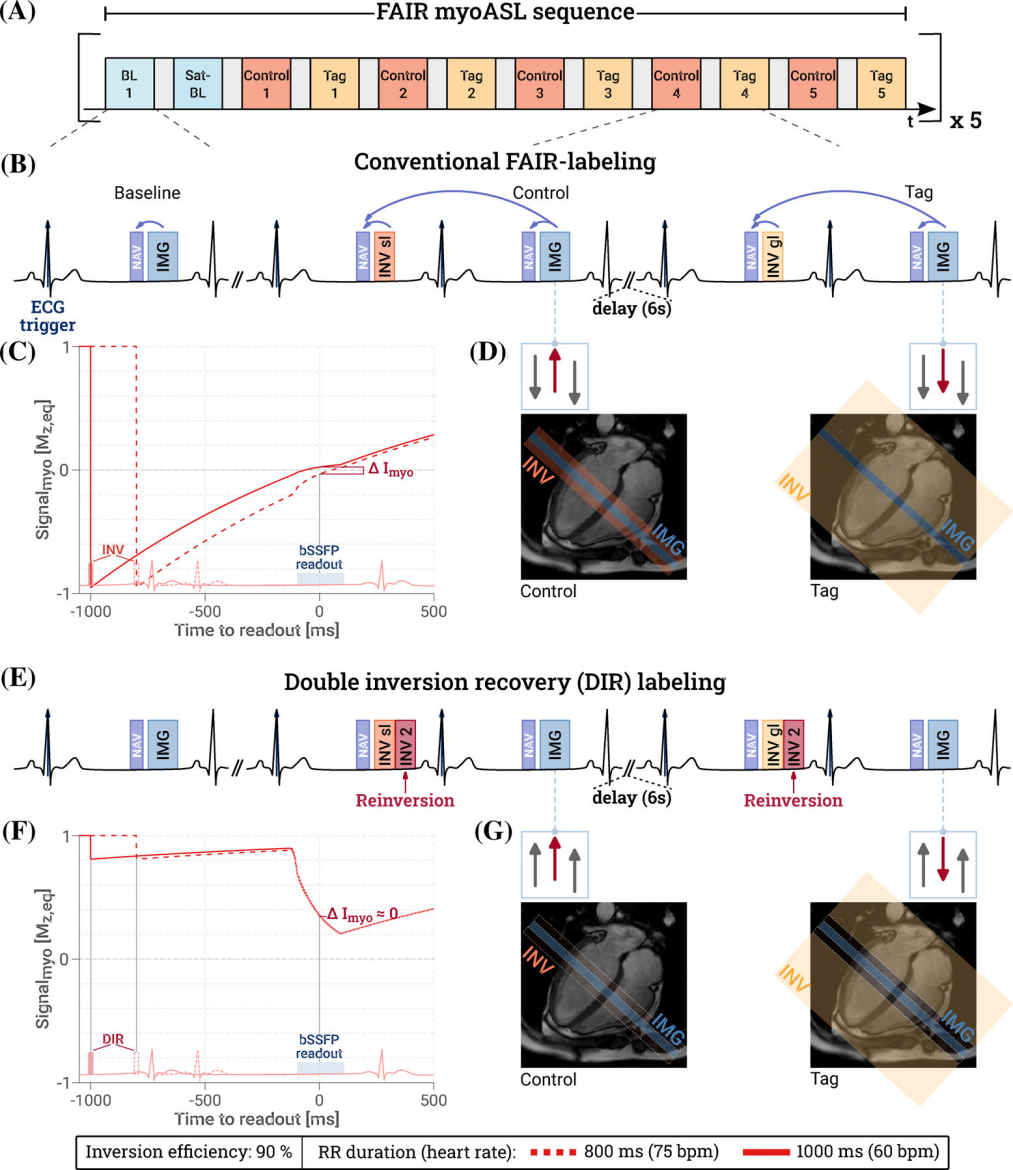
(A) Diagram of the double ECG‐gated, respiratory‐navigated FAIR myocardial ASL sequence. (B) After a selective/global inversion pulse, control/tag images are acquired during mid‐diastole, with a 6 s delay between images. Images are acquired only if both the current and preceding navigator are accepted. (E) For Double Inversion Recovery (DIR) preparations, a reinversion pulse is added right after the FAIR‐labeling pulses. The imaging volume (blue) and corresponding inversion slabs (orange/yellow) are illustrated for control and tag settings in (D) conventional FAIR and (G) DIR with slice‐selective reinversion. With DIR, the myocardial signal (gray) is restored, while the in‐flowing blood signal (red) and thus the perfusion constant remain the same as with FAIR‐labeling. (C,F) Residual myocardial signal is substantially reduced in the presence of heart rate variations when using DIR compared with single inversion.

The proposed method builds on a double‐ECG gated FAIR‐myoASL sequence,^14^, ^15^ as illustrated in Figure 1. A delay of 6 s is added between consecutive control and tag images to allow for sufficient magnetization recovery. In this sequence, the inversion pulse as well as the imaging readout are triggered to the respective diastolic phases of two subsequent heartbeats. Thus, the TI depends on the duration of the RR interval and is subject to change with HR variations. With a modified Buxton's General Kinetic Model (GKM),^16^ as typically used in myoASL,^9^, ^10^, ^14^, ^17^ the myocardial blood flow can be quantified as

(1) $$MBF=\frac{\lambda (I_{C}-I_{T})}{\delta_{inv}I_{BL}TIe^{\frac{-TI}{T_{1,B}}}},$$

with control ($I_{C}$) and tag ($I_{T}$) image signal, baseline signal $I_{BL}$ as a surrogate for equilibrium magnetization $M_{0}$, blood‐water partition coefficient λ=1.0 mL/g,^18^, ^19^ inversion factor $\delta_{inv}=2$, and blood $T_{1}$ relaxation time $T_{1,B}$. The perfusion‐weighted signal and corresponding noise terms for FAIR‐myoASL are calculated in Appendix A. As apparent from Equation (A6), a nonzero PN component arises in the presence of TI discrepancies due to HR variations.

### 2.2 Double Inversion Recovery preparation

To mitigate the effect of variable signal recovery, in this work, a second inversion pulse is applied immediately after the FAIR‐labeling with the shortest delay allowed by the transmit hardware (150 μs). The effectiveness of this modification is studied for slice‐selective and nonselective reinversion pulses. These are referred to as selective and nonselective DIR‐labeling, respectively. As illustrated in Figure 1, the reinversion ensures near‐complete recovery of the stationary myocardial tissue during TI. Thus, even in the presence of a varying TI, the myocardial signal cancels out more effectively in the subtraction of the control and tag image. A detailed description of the perfusion‐weighted signal resulting from selective and nonselective reinversion is provided in Appendix A (Equations A10 and A13).

### 2.3 Simulation experiments

Numerical Bloch simulations were performed to study the efficacy and bias of DIR‐labeling with different inversion efficiency. To that end, perfusion values were estimated from simulated myoASL signal with conventional FAIR‐labeling and DIR‐labeling for a range of inversion efficiencies (85–100%). General simulation parameters were chosen to match the phantom experiments and are summarized in Table 1. Imaging readout was simulated based on a single RF pulse and without ramp‐up pulses to eliminate its potential influence on the MBF values.^15^ The corresponding signal models for FAIR as well as selective and nonselective DIR‐labeling are detailed in Appendix  A (Equations (A1), (A2), (A3), A8, A9, A11, and A12, respectively). A blood‐volume fraction $V_{B}$ of 0.14^20^ and a blood replacement (in‐flow) rate $f_{in}$ of 0.29 1/s^21^, ^22^ were simulated. Together with λ = 1.0 mL/g, this results in an effective MBF input value of $f_{in}\cdot \lambda \cdot V_{B}$ = 2.4 mL/g/min. Other physiological parameters were simulated as HR 60 bpm, blood $T_{1}$/$T_{2}$ relaxation times at 3 T of 2090 ms/160 ms, and myocardial $T_{1}$/$T_{2}$ relaxation times of 1370 ms/60 ms,^23^, ^24^ again to match the relaxation times in phantom.

**TABLE 1:** Sequence parameters of the FAIR‐myoASL sequence for numerical simulations, phantom and in vivo measurements with conventional FAIR and DIR‐labeling.

Experiment	FA (°)	TE/TR (ms)	Matrix size	FOV (mm 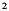 )	Voxel size (mm 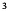 )	PF/GRAPPA
Simulations	70	1.55/3.1	1 × 1	n.a.	n.a.	n.a.
Phantom	70	1.68/3.36	176 × 176	300 × 300	1.7 × 1.7 × 8.0	6/8/*R* = 2
In vivo	70	1.63/3.26	170 × 208	341 × 291	1.9 × 1.9 × 8.0	6/8/*R* = 2

Abbreviations: GRAPPA, Generalized Auto‐Calibrating Partially Parallel Acquisition; PF, Partial Fourier.

To assess the TI variability‐related PN, the myoASL signal was simulated for six control–tag pairs with randomly varying RR interval durations for all three labeling strategies. Zero‐mean Gaussian noise was added to the simulated RR interval duration with a standard deviation $\sigma_{RR}$ ranging from 0 ms to 150 ms (CoV: 0%–15%), in line with HR variabilities commonly observed in vivo.^25^, ^26^, ^27^ Additional thermal noise was modeled as constant, zero‐mean Gaussian noise. The standard deviation of the thermal noise was chosen such that the ratio of physiological to thermal noise ranged between 0 and 6 across the range of simulated RR variability ($\sigma_{RR}$).^10^ All simulations were repeated n=1000 times per setting to determine an average $SNR\pm \sigma_{SNR}$ as a function of HR variability. The correlation of the PN with $\sigma_{RR}$ was evaluated using Spearman's correlation. The simulated MBF values were compared across the three labeling strategies using analysis of variance (ANOVA) for group‐wise comparison, followed by pair‐wise comparison using t‐tests. A significance level of 0.05 was used in all statistical tests. Slope and intercept values were obtained from a linear regression of simulated PN values and are reported with a 95% confidence interval.

In addition to the simulation experiments, a phantom study was conducted to evaluate the influence of DIR‐labeling on the MBF values and PN of FAIR‐myoASL. Details are provided in the Phantom Experiments section of the Supporting Information, and the specific sequence parameters are listed in Table 1.

### 2.4 In vivo experiments

All imaging was performed at 3 T (Magnetom Skyra, Siemens Healthineers, Erlangen, Germany). Adiabatic inversion labeling was achieved using hyperbolic secant pulses (duration: 10.2 ms). For reinversion in DIR‐preparations, an identical pulse with inverted pulse phase was used.

This study was approved by the local institutional review board. Six healthy volunteers (4 male, 2 female, 29.5 ± 2.1 years) without history or current symptoms of cardiovascular disease were included in this study. All subjects gave written and informed consent before examination, and the study was approved by the institutional ethics committee (Ethics Committee II, Heidelberg University, 2022‐541‐AF‐5). In vivo myoASL imaging was performed at rest in free‐breathing acquisitions with a dual‐respiratory navigator.^28^ Dual respiratory navigation was performed by playing a pencil‐beam navigator placed at the liver dome for both, heartbeats with labeling pulses and heartbeats with image readouts. Images were acquired only when both consecutive navigators were within the predefined acceptance window, to improve alignment of the imaging slices and the selective inversion slab.

Conventional myoASL with single FAIR‐labeling, and slice/nonselective DIR‐prepared myoASL were acquired in vivo, with sequence parameters as given in Table 1. Each of the three sequences comprised five control–tag pairs and two baseline images, as shown in Figure 1. Although both conventional and saturation‐prepared baseline images were acquired, the saturation‐baseline correction was ultimately not used for quantification as its effectiveness is limited with bSSFP readouts.^15^ All scans were repeated five times in randomized order to assess repeatability. Additionally, a Modified Look‐Locker Inversion recovery (MOLLI)^29^ $T_{1}$‐mapping sequence was acquired to determine individual blood $T_{1}$ relaxation times for MBF calculation.^15^ All scans were corrected for residual motion using a learning‐free neural‐network‐based group‐wise image registration method.^30^, ^31^ Manual segmentation of the left ventricular blood pool and myocardium was performed using the baseline images. Next, MBF values were calculated in a pixel‐wise manner using Buxton's GKM.^16^ Individual blood $T_{1}$ relaxation times were measured and used in the quantification to mitigate the HR dependence of MBF values.^15^ The corresponding PN values were calculated in a pixel‐wise manner as previously described^10^, ^11^, ^15^:

(2) $$PN=\frac{\sigma_{MBF}}{\sqrt{N_{CT}}}$$

where $\sigma_{MBF}$ denotes the standard deviation of MBF values across the $N_{CT}$ = 5 control–tag pairs acquired in each measurement. The relative SNR gain compared to conventional FAIR‐myoASL was determined for both DIR‐strategies as:

(3) $$\Delta SNR=\frac{SNR_{DIR}}{SNR_{conv}}=\frac{\mu_{MBF_{DIR}}}{\sigma_{MBF_{DIR}}}\cdot {\left(\frac{\mu_{MBF_{conv}}}{\sigma_{MBF_{conv}}}\right)}^{-1}$$

with mean $\mu_{MBF}$ and standard deviation $\sigma_{MBF}$ of the MBF obtained across the five control–tag pairs acquired in each repetition. The in vivo RR variability, PN and SNR gain were compared across the labeling strategies using a Friedman test for group‐wise comparison, followed by a Wilcoxon signed‐rank test for pair‐wise comparison. The correlation of the mean PN and RR variability ($\sigma_{RR}$) across the five sequence repetitions was evaluated using Spearman's correlation. Linear regression analysis between PN and $\sigma_{RR}$ was performed to test for dependence on RR variability. For FAIR‐labeling, one data point (subject 3, sequence repetition 2) was excluded due to extremely high RR variability ($\sigma_{RR}>$500 ms), likely caused by ECG‐mistriggering or severe subject movement during the measurement. A significance level of 0.05 was used in all statistical tests.

## 3 RESULTS

### 3.1 Simulation results

**FIGURE 2:**
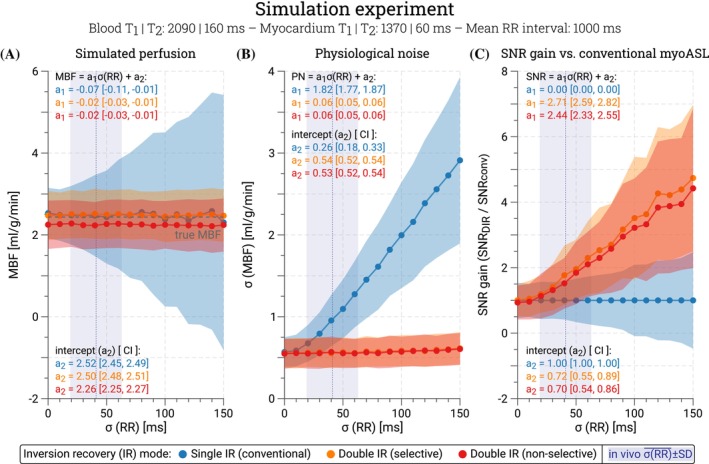
Simulated (A) myocardial blood flow (MBF), (B) physiological noise (PN), and (C) relative SNR gain for an inversion efficiency of 90%. Data are shown as a function of the heart rate (HR) variability for conventional FAIR (blue), selective (orange), and nonselective (red) double inversion recovery (DIR) labeling. Linear regression slopes ($a_{1}$, in units of $10\;s^{-1}$) and intercepts ($a_{2}$) are reported with the corresponding 95% confidence interval (CI). The average HR variability ± SD as observed in our in vivo study is highlighted in purple. MBF values were comparable between conventional FAIR and selective DIR‐labeling, while nonselective DIR‐labeling yielded lower perfusion values due to incomplete control signal recovery. When DIR‐labeling was used, heart‐rate‐induced PN was fully eliminated. Within the range of in vivo HR variability, this resulted in an average 1.76/1.52‐fold (slice‐selective/nonselective) SNR gain compared to conventional FAIR.

The effect of a variable HR on simulated myoASL measurements using conventional FAIR and DIR‐labeling is illustrated in Figure 2 for a simulated inversion efficiency of 90%. Simulated myoASL‐perfusion values were generally comparable between conventional FAIR (2.44 ± 0.10 mL/g/min), selective DIR (2.48 ± 0.02 mL/g/min), and nonselective DIR‐labeling (2.24 ± 0.02 mL/g/min). For low inversion efficiencies, nonselective DIR‐labeling showed significantly lower MBF values compared to conventional FAIR (p<0.001) and selective DIR‐labeling (p<0.001). This difference diminished for higher inversion efficiencies, as shown in Supporting Information Figure S1 and Supporting Information Table S1. At 90% inversion efficiency and a mean in vivo HR variability of 41 ms, a simulated PN of 0.95 ± 0.31 mL/g/min was observed for conventional FAIR. With DIR‐labeling, the PN was reduced by 42% for selective (0.55 ± 0.18 mL/g/min) and by 40% for nonselective reinversions (0.57 ± 0.19 mL/g/min), respectively. For all methods, the simulated PN increased with increasing HR variability (Supporting Information Figure S2, Supporting Information Table S2). However, for DIR‐labeling, this effect was greatly alleviated, especially for higher inversion efficiency (regression slope [CI]: 0.24 [0.22, 0.26], $R^{2}$ = 1.0 at 85%, to 0.01 [0.00, 0.01], $R^{2}$ = 0.58/$R^{2}$ = 0.42 (selective/nonselective DIR) at 100% inversion efficiency).

**FIGURE 3:**
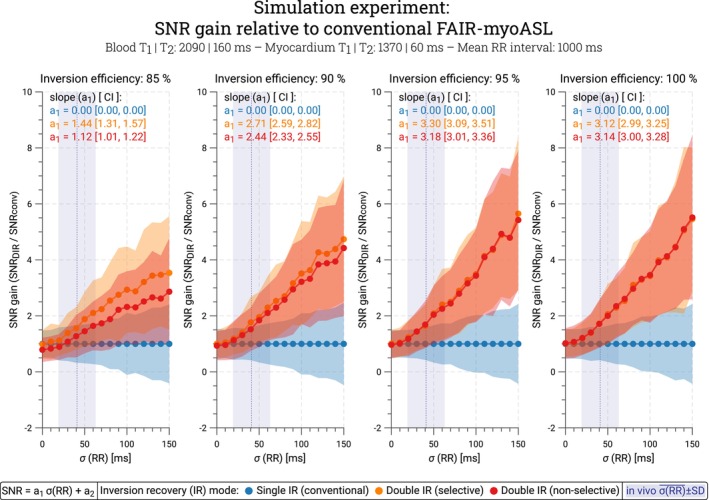
Relative SNR gain with double inversion recovery (orange, red) compared to a conventional FAIR‐myoASL sequence (blue) in simulation experiments. The SNR gain is shown as a function of the simulated heart rate variability that models the physiological noise. Linear regression slopes ($a_{1}$) are reported with the corresponding 95% confidence interval (CI) in units of $10\;s^{-1}$. The average HR variability ±SD as observed in our in vivo study is highlighted in purple. The SNR gain increases with increasing simulated ratio of physiological and thermal noise as well as with higher inversion efficiency ($0.98<R^{2}<1.0/0.97<R^{2}<0.99$, slope: 1.44–3.30/1.12–3.18 for selective/nonselective DIR). For a mean in vivo HR variability of 41 ms, an average SNR gain between 1.58/1.29 and 1.76/1.75 times was achieved with selective/nonselective DIR‐labeling.

Consequently, greater inversion efficiencies led to higher SNR gain compared to conventional FAIR for both selective ($R^{2}$ = 1.0, 1.44 [1.31, 1.57] < slope [CI] < 3.30 [3.09, 3.51]) and nonselective DIR‐labeling ($R^{2}$ = 1.0, 1.12 [1.01, 1.22] < slope [CI] < 3.18 [3.01, 3.36]), as illustrated in Figure 3 (Supporting Information Table S3). At 90% inversion efficiency, DIR‐labeling resulted in an SNR gain of 1.79 ± 0.90 with selective and 1.55 ± 0.77 with nonselective reinversions, within the range of HR variability observed in vivo. As the level of thermal noise increased, the overall SNR gain achieved with DIR‐labeling decreased. In the absence of thermal noise, the SNR gain remained constant across the range of simulated RR variability (Supporting Information Figure S3).

The results of the phantom study show similar trends, as illustrated in Supporting Information Figure S4. Across the range of simulated HR variability ($\sigma_{RR}$), the average phantom MBF values remained largely constant at around 3.3 mL/g/min for all three myoASL sequences. While the PN increased with higher HR variability in all three sequences, both selective and nonselective DIR‐labeling (slope [CI]: 0.04 [0.03, 0.06]) mitigated this effect compared to conventional FAIR (slope [CI]: 0.74 [0.63, 0.84]). For a $\sigma_{RR}$ corresponding to the mean HR variability in vivo, this led to a relative SNR gain of 2.91 ± 0.52 for selective and 3.56 ± 0.53 for nonselective DIR‐labeling. Similar to the simulation results, the SNR gain in phantom remained constant in the absence of thermal noise and decreased with increasing thermal noise levels across the whole range of $\sigma_{RR}$ (Supporting Information Figure S3).

### 3.2 In vivo results

**FIGURE 4:**
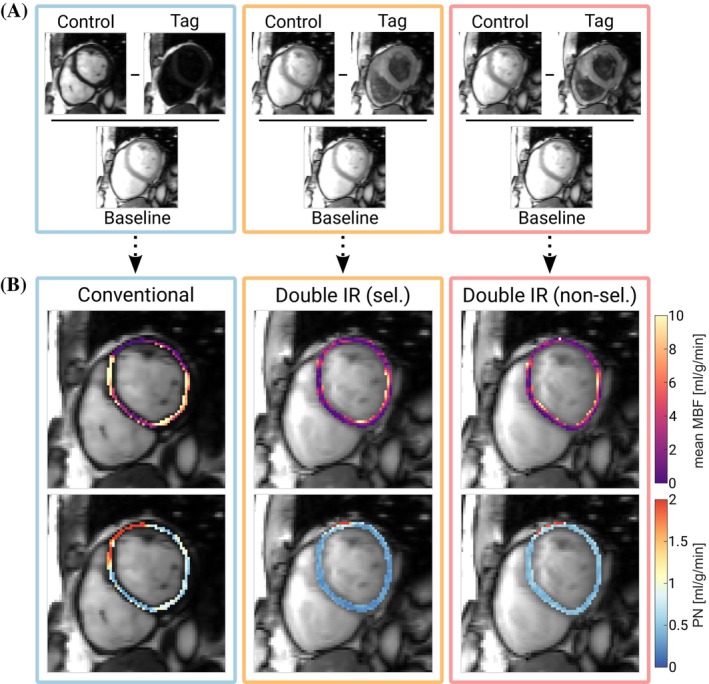
(A) Representative control, tag, and baseline images for conventional FAIR‐myoASL (blue) as well as double inversion recovery (DIR) labeling with selective (orange) and nonselective (red) reinversion pulses. (B) Resulting in vivo perfusion (top) and physiological noise (PN) maps (bottom). Improved map quality and lower PN levels were achieved with DIR‐labeling compared to the conventional sequence.

**FIGURE 5:**
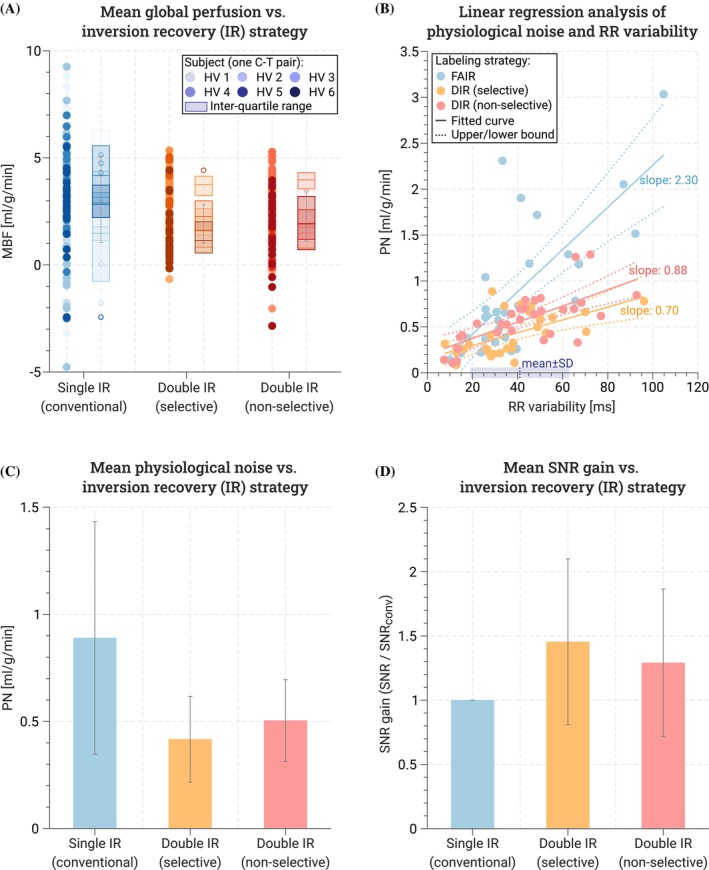
(A) In vivo global myoASL‐MBF for all acquired control–tag pairs. Values for the six subjects are depicted with lighter and darker shades of the colors corresponding to the three acquired sequences. (B) The average RR variability ±SD across all sequences and measurements is highlighted in purple. Compared to FAIR‐labeling (slope: 2.30, p< 0.05), linear regression slopes of physiological noise (PN) as a function of RR variability were reduced more than three‐fold with selective (0.70, p< 0.05) and 2.5‐fold with nonselective DIR‐labeling (0.88, p< 0.05). (C) When double inversion recovery (DIR) preparations were used, the average PN was reduced by 53% for selective and 44% for nonselective reinversion pulses compared with conventional myoASL. (d) Relative to conventional myoASL the SNR increased 1.47 ± 0.63 times and 1.32 ± 0.57 with selective and nonselective DIR‐preparations, respectively.

Figure 4 shows the control, tag, and baseline images obtained in vivo alongside the resulting MBF and PN maps for one subject. In visual assessment, perfusion maps obtained with DIR‐labeling showed improved image quality compared with conventional FAIR‐myoASL. Moreover, PN maps showed overall lower values for DIR‐labeling compared with conventional FAIR, particularly in the inferior and inferoseptal regions. For each acquired myoASL sequence, the mean global MBF is shown in Figure 5 per acquired control–tag pair in all subjects. Across all subjects, the average blood $T_{1}$ relaxation time was 1826 ± 51 ms [1725–1862 ms], and the average RR variability was 41 ± 22 ms [7–105 ms]. No significant difference in average subject RR variability was found between the three sequences (FAIR: 41 ± 24 ms, selective DIR: 38 ± 19 ms, nonselective DIR: 44 ± 22 ms, p > 0.22).

Using conventional FAIR‐labeling, the in vivo mean global MBF ± SD was 2.88 ± 1.06 mL/g/min across all subjects. With DIR‐preparations, the mean MBF ± SD was 2.04 ± 0.98 mL/g/min for selective reinversion pulses and 2.22 ± 0.94 mL/g/min for nonselective reinversion pulses. In addition, Figure 5 shows the corresponding PN and SNR gain relative to conventional FAIR‐labeling averaged across all subjects and the five repetitions of each sequence. In group‐wise comparison, the PN showed significant differences among the three labeling strategies (p<0.05). The mean PN ± SD across all subjects was 0.89 ± 0.54 mL/g/min in conventional FAIR‐labeling and was reduced on average by 53% (p<0.05) with selective and by 44% (p=0.09) with nonselective DIR‐preparations, respectively. With FAIR‐labeling, the mean subject PN showed a statistically significant, strong correlation with RR variability ($\rho_{Spearman}$ = 0.75, p< 0.05) and a regression slope of 2.30 (CI: [1.48, 3.12], p< 0.05). For selective and nonselective DIR‐labeling, statistically significant but more moderate correlations were observed. However, substantially lower regression slopes were observed compared to conventional FAIR, for both selective (slope: 0.70, CI: [0.33, 1.07], p< 0.05) and nonselective reinversions (slope: 0.88, CI: [0.52, 1.24], p< 0.05). Detailed correlation and linear regression parameters are summarized in Supporting Information Tables S4 and S5. The use of DIR‐labeling resulted in a trend of substantially higher SNR (SNR gain across all subjects: 1.47 ± 0.63 selective‐DIR, 1.32 ± 0.57 nonselective DIR). However, the difference was not found to be statistically significant in the small cohort (p=0.06 selective, and p=0.22 nonselective).

## 4 DISCUSSION

In this study, we investigated the use of Double Inversion Recovery labeling in double ECG‐gated FAIR‐myoASL to reduce physiological noise (PN) related to HR variations. Reinversion immediately after the FAIR preparation causes near‐complete signal recovery of static tissue. This mitigates differences in the signal relaxation between acquisitions and facilitates effective cancellation of the background signal in the subtraction of control and tag images. In simulations and phantoms, DIR‐labeling using either a selective or nonselective reinversion pulse demonstrated reduced PN and increased SNR compared to conventional FAIR‐labeling. In vivo, the proposed DIR approaches yielded comparable perfusion values to conventional FAIR‐myoASL, and a trend of reduced PN.

In simulation and phantom experiments, the observed myocardial blood flow (MBF) values were largely comparable between single FAIR‐labeling and DIR‐labeling across the range of simulated HR variability. In phantom, a residual bias in MBF values relative to the input perfusion rate was observed across all sequences, which can be attributed to the effect of the imaging readout.^15^ In vivo, mean global MBF for all three labeling strategies was in line with perfusion values reported in PET literature (0.74–2.43 mL/g/min).^32^ Compared to reported perfusion values from first‐pass perfusion CMR (0.62–1.24 mL/g/min),^33^, ^34^ the MBF values from DIR‐preparations were slightly elevated, while those obtained with conventional FAIR were generally above the reported range. However, MBF estimates from all three investigated sequences agreed with perfusion values reported in previous studies using myoASL (0.7–2.7 mL/g/min).^10^, ^14^, ^17^, ^35^

Our phantom and simulation results indicate that PN caused by TI variation accounts for a substantial fraction of the overall measurement noise. Considering an expected thermal noise of 0.55 mL/g/min, a simulated RR variability of 50 ms resulted in a net PN of 0.58 mL/g/min, representing approximately 50% of the total measurement noise. In healthy adults, the HR variability can be expected to be commonly around 100 ms.^25^, ^26^ Thus, our results underscore that the TI variations are expected to be the single largest contributor to PN in double ECG‐gated FAIR‐myoASL, which is in line with previous findings.^10^

In agreement with the simulation and phantom results, a significant positive correlation of PN and RR variability was found in vivo, confirming the considerable influence of a variable HR on the PN for conventional FAIR‐labeling. However, the use of the proposed DIR‐preparations substantially mitigates the confounding effect of variable inversion times. Consequently, more moderate correlation of in vivo PN and the RR variability was found for both selective and nonselective reinversions. Furthermore, an approximately threefold reduction in the regression slope indicates improved resilience to RR variability.

The PN levels observed in the in vivo experiments were comparable to the range reported in earlier myoASL studies.^10^, ^14^, ^17^, ^35^ Compared with conventional FAIR‐myoASL, the PN was reduced on average by 53% with selective and 44% with nonselective DIR‐preparations. In addition to reducing the overall PN through suppression of TI‐related signal fluctuations, DIR‐labeling also led to a more homogeneous spatial distribution of PN across the myocardium. This heterogeneity can be related to residual (through‐plane) motion, which has a more pronounced impact in FAIR‐labeling due to the stronger influence of inversion profiles compared with DIR. The mean ratio of PN to MBF of 35% in conventional acquisitions was significantly reduced to 23% when selective reinversion was applied. With nonselective reinversions, this ratio was reduced by 27%, although this trend did not reach statistical significance in the small sample cohort. Nonetheless, compared with an anticipated reduction in stress MBF of about 55% in CAD,^34^, ^36^, ^37^ the proposed DIR methods can potentially be instrumental in improving the detection of perfusion defects with FAIR‐myoASL.

The choice of slice‐selective or nonselective reinversion pulses in the DIR‐preparation affected the estimated MBF and associated PN. The simulation results showed that nonselective reinversion imparts bias at low inversion efficiencies, leading to lower MBF compared with slice‐selective reinversion. With nonselective DIR‐preparations, the blood entering the imaging slice is inverted twice. Hence, the control signal in blood is a function of the inversion/reinversion efficiency. This is in contrast to single FAIR‐labeling or selective DIR‐labeling, where inflowing blood remains unaltered. Consequently, nonselective DIR‐preparations introduce an additional, HR‐dependent bias to the perfusion‐weighted signal, which amplifies the PN compared to their slice‐selective counterpart and the conventional FAIR‐labeling. In simulations, the efficiency of the inversion/reinversion block was assumed to be the inversion efficiency of a single pulse, to the power of two. This can provide an upper bound and reflects the situation of complete spoiling of the transverse magnetization between the two pulses. However, due to the symmetry of the adiabatic sweep, inversion/reinversion blocks can partially compensate for imperfections introduced due to $B_{0}$ and $B_{1}$ field inhomogeneities.^38^ Thus, the overall efficiency of the inversion/reinversion block is typically higher than the squared efficiency of the constituent pulses. Nonetheless, slice‐selective DIR‐preparations demonstrated superior SNR performance, particularly at low inversion efficiencies. Accordingly, when applied in vivo, slice‐selective DIR‐labeling led to lower PN levels and more SNR improvement than nonselective DIR‐labeling. Lower perfusion values were observed with slice‐selective compared to nonselective reinversions. However, this can likely be attributed to reduced nonzero‐mean contributions to the PN.^39^

The proposed DIR‐preparations specifically target contributions to PN arising from HR‐induced TI variability. In brain ASL, background suppression techniques are widely used to reduce unwanted signal fluctuations in the ASL images.^40^ These typically involve a combination of presaturation pulses before blood labeling to compensate for imperfect slice profiles, and multiple inversion pulses between labeling and image acquisition.^41^, ^42^ These additional inversion pulses are carefully timed to null static tissue signal while minimally affecting the ASL signal itself.^43^, ^44^ Similar approaches have also been proposed for myoASL, where additional nonselective inversions after the FAIR‐labeling null the overall myocardial signal at the time of readout.^45^, ^46^ While this approach can mitigate fluctuations of the myocardial background signal, it remains sensitive to variable recovery times due to HR variability—unlike brain ASL, where the additional inversions can be timed more precisely. Importantly, the proposed DIR approach differs from these techniques with regard to their respective objectives. While background suppression is designed to null the static tissue signal at image readout, ideally eliminating it before subtraction, DIR aims to restore it to a similar level in both control and tag settings. Thereby, the static tissue signal can be canceled out more effectively during subtraction regardless of the specific TI.

Previous studies have also proposed the addition of presaturation pulses immediately before the FAIR‐labeling in cardiac settings.^17^, ^47^ By facilitating a higher degree of signal recovery of myocardial background signal during TI, this approach addresses physiological noise stemming from TI variability similar to the DIR‐labeling proposed in this work. However, presaturation also affects the inflowing blood and lowers the perfusion‐related signal contribution. Furthermore, the effectiveness of noise suppression with presaturation depends on the efficiency of the saturation pulses, which are usually applied spatially selectively. Additional reinversion pulses, on the other hand, can achieve near‐complete signal recovery within a single heartbeat, even in the presence of imperfect inversion/reinversion efficiency and without compromising the perfusion signal. Our results indicate that successfully suppressing signal fluctuations caused by TI variability greatly reduces PN. Thus, the proposed DIR‐labeling technique proved very effective in achieving an overall higher SNR.

Overall, the SNR gain achieved in vivo was slightly lower than the values obtained from simulation and, particularly, phantom experiments. As these do not fully capture the complexity of myocardial perfusion imaging, the substantially higher SNR gains observed at simulated HR variabilities above approximately 120 ms should be interpreted as upper bounds under idealized conditions. More realistic modeling of PN, including flow‐phantom studies, is warranted to better reflect clinical settings. Nevertheless, the simulation and phantom results in this study suggest that DIR‐labeling may lead to greater gains in SNR in scenarios with pronounced HR variability, such as in patients with arrhythmia.^48^, ^49^

It should be noted that the SNR gain achieved with DIR‐labeling strongly depends on the relative contribution of physiological versus thermal noise. Since DIR mitigates PN by suppressing signal fluctuations due to TI variability, it does not reduce thermal noise in the measurement. As a result, higher ratios of physiological to thermal noise lead to a more pronounced overall SNR improvement with DIR compared to FAIR‐labeling. Another implication is that differences in RR variability between labeling strategies may confound the observed SNR gain. While there was no significant difference among the RR variabilities observed for the three sequences in this study, potential differences in RR variability warrant careful consideration in future studies.

Another approach to reduce the effect of TI variability on perfusion quantification with Buxton's General Kinetic Model (GKM)^16^ is to incorporate separate TI values for the weighting of the control and tag image within the quantification model.^13^ This approach can be applied to various myoASL techniques, provided the individual TI values for each acquisition are recorded. However, our previous results indicate that this quantification method yields only a marginal reduction in MBF variation relative to employing an averaged TI within the GKM.^15^ Recently, perfusion quantification based on three‐parameter fitting models has been shown to provide higher temporal SNR and improved reproducibility compared to TI correction approaches.^11^ However, both TI correction and fitting models predominantly account for the recovery of blood signal, since Buxton's GKM considers exclusively the $T_{1}$ relaxation in blood. The compensation of signal fluctuation in the myocardium remains incomplete due to the difference in $T_{1}$ values between myocardium and blood at 3 T.^24^ Nonetheless, these TI evaluation approaches are complementary to many acquisition‐based strategies. Thus, they can also be combined with the proposed DIR‐labeling to further reduce the sensitivity of double ECG‐gated FAIR‐myoASL to a varying HR.

The method in this study relies on the acquisition of multiple control–tag image pairs to ensure robust perfusion quantification^10^, ^11^, ^14^, ^50^. However, the perfusion sensitivity in FAIR is predominantly encoded within the control image, while the tag image primarily serves to suppress background signal from inverted blood and myocardial tissue. Given that TI variations are the main contributor to PN, acquiring fewer—or even a single—tag image per sequence may suffice when combined with DIR‐labeling, which mitigates TI‐related signal fluctuations. This could enable more time‐efficient acquisitions without a substantial loss in SNR.

The dual respiratory navigation used in this study ensures that images are acquired only upon successful inversion, but may prolong acquisition times due to its reliance on two consecutive heartbeats. While this is particularly relevant for slice‐selective preparations, nonselective preparations are less susceptible to cardiac motion. Alternative respiratory gating schemes, in which dual gating is only applied for slice‐selective inversion pulses, could therefore reduce scan durations. This may be especially beneficial in clinical settings where the observed breathing pattern is less regular and time efficiency is critical.

Although no statistical significance was achieved for nonselective DIR in vivo, selective DIR demonstrated significant reductions in PN and a clear trend toward improved SNR. Thus, a study focusing on selective DIR in a larger cohort could provide a deeper understanding of the robustness of DIR‐labeling and allow for improved statistical comparison to conventional FAIR‐myoASL. Moreover, MOLLI $T_{1}$‐mapping,^29^ from which blood $T_{1}$ values were obtained for in vivo MBF quantification, is known to underestimate $T_{1}$ values. Alternatively, saturation‐based methods, such as saturation recovery single‐shot acquisition (SASHA),^51^ could offer a solution to this potential confounding factor, albeit its availability on clinically available MR systems remains limited compared to MOLLI. The current study investigated the use of DIR‐prepared FAIR‐myoASL in healthy subjects at rest only. Particularly in view of translation to the clinic, in‐depth evaluation of the proposed method at rest and during stress is required. Especially patient populations presenting with cardiac arrhythmias as comorbidity warrant further research to assess the PN reduction due to HR variability in a clinical setting.^48^, ^49^

## 5 CONCLUSION

Heart‐rate‐induced variability in the inversion time is a major source of physiological noise in myocardial ASL. This can be mitigated by adding a reinversion pulse immediately after the FAIR‐labeling, allowing for more effective cancellation of the myocardial background signal in the presence of a varying HR. Our results show that using such Double Inversion Recovery preparations substantially reduced the physiological noise in phantom and in vivo, and mitigated the sensitivity to HR variations. Improving the precision of myoASL‐based perfusion measurements this way may prospectively contribute to a wider clinical adoption of myocardial ASL.

## CONFLICT OF INTEREST STATEMENT

The authors declare no potential conflict of interests.

## FUNDING INFORMATION

This work was supported by ZonMW Off‐Road, Grant/Award Number: 04510011910073; the European Research Council, Grant/Award Number: 101078711; Nederlandse Hartstichting, Grant/Award Number: 03‐004‐2022‐0079; Nederlandse Organisatie voor Wetenschappelijk Onderzoek, Grant/Award Number: STU.019.024; and 4TU Precision Medicine program supported by High Tech for a Sustainable Future. MBI received funding through the Landesgraduiertenförderung Baden‐Württemberg.

## Supporting information


**Data S1:**Supporting Information.

## APPENDIX A PERFUSION‐WEIGHTED SIGNAL IN DOUBLE ECG‐GATED MYOCARDIAL ASL

### A1 Conventional FAIR‐labeling

The myocardial blood flow (MBF) can be estimated from FAIR‐myoASL images using Buxton's General Kinetic Model (GKM)^16^ (see Equation 1). Here the MBF is directly proportional to the difference of control ($I_{C}$) and tag signal ($I_{T}$). For conventional FAIR‐labeling, the control, tag and baseline signal ($I_{BL})$ can be expressed as

(A1)
$$\begin{array}{ll} I_{C} & =V_{M}(A_{M}x_{M,C}^{-}+B_{M})+\\ & \;+V_{B}(f_{in}(A_{B}x_{B,C}^{+}+B_{B})+\\ & \;+(1-f_{in})(A_{B}x_{B,C}^{-}+B_{B})), \end{array}$$

(A2)
$$\begin{array}{cc} I_{T} & =V_{M}(A_{M}x_{M,T}^{-}+B_{M})+\\ & \;+V_{B}(A_{B}x_{B,T}^{-}+B_{B}), \end{array}$$

(A3)
$$\begin{array}{ll} I_{BL} & =V_{M}(A_{M}x_{M,BL}^{+}+B_{M})+\\ & \;+V_{B}(A_{B}x_{B,BL}^{+}+B_{B}), \end{array}$$

with blood and myocardial signal contributions weighted by the blood‐volume fraction $V_{B}=1-V_{M}$ and myocardial‐volume fraction $V_{M}$. The acquisition coefficients $A_{M}$ and $A_{B}$ depend on the respective relaxation times in the myocardium and blood as well as the acquisition parameters of the myoASL image readout.^52^ The initial magnetization x prior to readout can be given as

(A4)
$$\begin{array}{ll} x^{+} & =M_{z,eq} \end{array}$$

(A5)
$$\begin{array}{ll} x^{-} & =M_{z,eq}\cdot (1-(1-\cos(\alpha_{inv}))e^{\frac{-TI}{T_{1}}}), \end{array}$$

for native and inversion‐prepared signals, respectively. The difference between control and tag signal can be decomposed as the sum of differences in background myocardium ($\Delta I_{M}$) and blood signal ($\Delta I_{B}$) and the blood‐flow‐related signal:

(A6)
$$\begin{array}{ll} \Delta I & =I_{C}-I_{T}=\\ & =V_{M}A_{M}(x_{M,C}^{-}-x_{M,T}^{-})\;+\;\{\text{I:}=\Delta I_{M}\\ & \;+V_{B}A_{B}(x_{B,C}^{-}-x_{B,T}^{-})\;+\;\{\text{II:}=\Delta I_{B}\\ & \;+V_{B}A_{B}f_{in}(x_{B,C}^{+}-x_{B,C}^{-}),\;\{\text{III:}\propto \text{MBF} \end{array}$$

The noise terms $\Delta I_{M}$ and $\Delta I_{B}$ from myocardial and blood contributions, respectively, depend on the difference in TI between control and tag image (ΔTI). Assuming that other sources of discrepancy between control and tag signal are negligible, $\Delta I_{M}$ and $\Delta I_{B}$ are zero only if ΔTI=0. If ΔTI≠0, however, $\Delta I_{M}$ and $\Delta I_{B}$ act as additional error terms in the calculation of perfusion values in the GKM using Equation (1). This is particularly relevant in double ECG‐gated myoASL, where both labeling and image readout are triggered to occur in the same cardiac phase, rendering the TI heart‐rate‐dependent.

### A2 Selective and nonselective DIR‐labeling

When slice‐selective reinversion pulses are applied, the magnetization of both static tissue and blood within the imaging volume is inverted twice. Hence, the initial magnetization prior to the readout with slice‐selective DIR‐preparations becomes:

(A7)
$$x^{\prime +}=M_{z,eq}\cdot (1-(1-\cos{\left(\alpha_{inv}\right)}^{2})e^{\frac{-TI}{T_{1}}})\text{,}$$

The signal of in‐flowing blood, however, is not affected by the additional slice‐selective pulse. The resulting control and tag signal for selective DIR can be given as

(A8)
$$\begin{array}{ll} I_{C}^{SS} & =V_{M}(A_{M}x_{M,C}^{\prime +}+B_{M})+\\ & \;+V_{B}(f_{in}(A_{B}x_{B,C}^{+}+B_{B})+\\ & \;+(1-f_{in})(A_{B}x_{B,C}^{\prime +}+B_{B})), \end{array}$$

(A9)
$$\begin{array}{ll} I_{T}^{SS} & =V_{M}(A_{M}x_{M,T}^{\prime +}+B_{M})+\\ & \;+V_{B}(f_{in}(A_{B}x_{B,C}^{-}+B_{B})+\\ & \;+(1-f_{in})(A_{B}x_{B,C}^{\prime +}+B_{B})). \end{array}$$

Based on this, the signal difference between control and tag image can be decomposed as

(A10)
$$\begin{array}{ll} \Delta I_{SS} & =I_{C}^{SS}-I_{T}^{SS}=\\ & =V_{M}A_{M}(x_{M,C}^{\prime +}-x_{M,T}^{\prime +})\;+\;\{\text{I:}=\Delta I_{M}^{\prime }\\ & \;+V_{B}A_{B}(x_{B,C}^{\prime +}-x_{B,T}^{\prime +})\;+\;\{\text{II:}=\Delta I_{B}^{\prime }\\ & \;+V_{B}A_{B}f_{in}(x_{B,C}^{+}-x_{B,C}^{-}).\;\{\text{III:}\propto \text{MBF} \end{array}$$

If, instead, nonselective reinversion pulses are used, spins within the imaging volume undergo double inversion, resulting in the same magnetization prior to readout as seen with slice‐selective reinversion pulses (Equation A7).

However, nonselective reinversion pulses also affect blood spins outside the imaging volume. A selective FAIR‐preparation combined with a nonselective reinversion causes inverted blood spins to flow into the imaging slice. With a nonselective FAIR‐preparation, the nonselective reinversion leads to in‐flowing blood spins being inverted twice. Thus, compared to a conventional FAIR‐preparation, nonselective DIR‐labeling leads to a swapping of control and tag images: The control setting is defined as nonselective FAIR‐labeling combined with nonselective reinversion. The tag setting then corresponds to the case of selective FAIR‐labeling followed by nonselective reinversion. With these considerations in mind, the control and tag image signal for nonselective DIR can be derived as

(A11)
$$\begin{array}{ll} I_{C}^{NS} & =V_{M}(A_{M}x_{M,C}^{\prime +}+B_{M})+\\ & \;+V_{B}(A_{B}x_{B,C}^{\prime +}+B_{B}), \end{array}$$

(A12)
$$\begin{array}{ll} I_{T}^{NS} & =V_{M}(A_{M}x_{M,T}^{\prime +}+B_{M})+\\ & \;+V_{B}(f_{in}(A_{B}x_{B,C}^{-}+B_{B})+\\ & \;+(1-f_{in})(A_{B}x_{B,C}^{\prime +}+B_{B})). \end{array}$$

Consequently, the difference between control and tag image for nonselective DIR yields

(A13)
$$\begin{array}{ll} \Delta I_{NS} & =I_{C}^{NS}-I_{T}^{NS}=\\ & =V_{M}A_{M}(x_{M,C}^{\prime +}-x_{M,T}^{\prime +})\;+\;\{\text{I:}=\Delta I_{M}^{\prime }\\ & \;+V_{B}A_{B}(x_{B,C}^{\prime +}-x_{B,T}^{\prime +})\;+\;\{\text{II:}=\Delta I_{B}^{\prime }\\ & \;+V_{B}A_{B}f_{in}(x_{B,C}^{\prime +}-x_{B,C}^{-}).\;\{\text{III:}\propto \text{MBF} \end{array}$$

While the noise terms $\Delta I_{M}^{\prime }$ and $\Delta I_{B}^{\prime }$ remain the same as for selective reinversion pulses (Equation A10), the perfusion‐weighted signal is different with nonselective DIR‐labeling. Instead of the native blood signal $x_{B}^{+}$, the perfusion‐weighted signal now contains the twice inverted blood signal $x_{B}^{\prime +}$. In consequence, an additional dependence on the inversion‐reinversion efficiency is introduced to the estimated perfusion values.
