# Supplementary material for: Myocardial Arterial Spin Labeling with Double Inversion Recovery for reduced physiological noise
Source: Magn Reson Med. 2025 Aug 11;94(6):2460–74. doi: 10.1002/mrm.70018 (PMC12501712; doi:10.1002/mrm.70018)
Supplement: Supplementary file 1 — Data S1:Supporting Information. [file MRM-94-2460-s001.pdf]

# SUPPLEMENTARY MATERIAL FOR:

Myocardial Arterial Spin Labeling with Double Inversion Recovery for Reduced Physiological Noise

Maša Božić-Iven[1, 2, 3], Stanislas Rapacchi[3], Yi Zhang[1], Qian Tao[1], Lothar R. Schad[2,3], and Sebastian Weingärtner[1]

[1]Magnetic Resonance Systems Lab, Department of Imaging Physics, Delft University of Technology, Delft, The Netherlands
[2]Computer Assisted Clinical Medicine, Medical Faculty Mannheim, Heidelberg University, Mannheim, Germany
[3]Mannheim Institute for Intelligent Systems in Medicine, Medical Faculty Mannheim, Heidelberg University, Mannheim, Germany
[4]Department of Diagnostic and Interventional Radiology, Lausanne University Hospital (CHUV), Lausanne, Switzerland

## INDEX OF SUPPLEMENTARY MATERIALS

Phantom experiments: Methods . . . 2
Numerical simulations: Results . . . 3
Phantom experiments: Results . . . 9
Correlation of PN with RR variability in vivo . . . 10

## INDEX OF SUPPLEMENTARY FIGURES AND TABLES

Figure S1 Simulated MBF with conventional FAIR and DIR-labeling vs. heart rate variability. 3

Figure S2 Simulated PN in conventional FAIR and DIR-labeling vs. heart rate variability. . . 4

Figure S3 Simulated and phantom SNR gain for varying ratio of physiological to thermal noise. 8

Figure S4 Phantom MBF, physiological noise and SNR gain vs. heart rate variability. . . . . 9

Table S1 Linear regression slope and intercept values of simulated MBF vs. RR variability. . 5

Table S2 Linear regression slope and intercept values of simulated PN vs. RR variability. . . 6

Table S3 Linear regression slope and intercept values of simulated SNR gain vs. RR variability. 7

Table S4 Spearman's correlation coefficients and p-values of in vivo PN vs. RR variability. . . 10

Table S5 Linear regression slope and intercept values of in vivo PN vs. RR variability. . . . . 10

# Phantom experiments: Methods

All imaging was performed at 3 T (Magnetom Skyra, Siemens Healthineers, Erlangen, Germany). Adiabatic inversion labeling was achieved using hyperbolic secant pulses (duration: 10.2 ms). For reinversion in DIR-preparations, an identical pulse with inverted pulse phase was used. Phantom experiments were conducted to evaluate the influence of DIR-labeling on the MBF values and PN of FAIR-myoASL. The phantom comprised 20 $NiCl_2$-doped agarose vials submerged in agarose gel, with $T_1$ relaxation times between 300 ms and 2500 ms and $T_2$ relaxation times between 40 ms and 170 ms. The two vials with $T_1/T_2$ relaxation times closest to that of myocardial tissue (1370 ms/60 ms) [1] and blood (2090 ms/160 ms)[4, 5], respectively, were selected for further processing. Conventional FAIR, selective DIR, and non-selective DIR myoASL were acquired, each comprising three control-tag image pairs and one pair of baseline images. Detailed sequence parameters can be found in Table 1 of the main manuscript. The phantom acquisitions were repeated for a range of simulated RR interval durations between 600 ms and 1200 ms in increments of 50 ms.

To mimic the in vivo HR variability in phantom acquisitions, a new data set was synthesized from the acquired phantom data. To that end, random RR interval durations were generated for each control-tag pair using a Gaussian distribution with mean values between 600 and 1200 ms and standard deviations, $\sigma_{RR}$, between 0 and 100 ms. The randomly generated RR duration was rounded to the closest acquired RR duration, and the synthetic image series was generated by randomly choosing images acquired with the respective RR duration. For each acquired labeling strategy and each level of HR variability, 100 image series were created this way, with each series containing 25 control-tag pairs and a baseline image. The control, tag, and baseline signals for the three sequences were synthesized from manually drawn ROIs in the selected vials. The corresponding signal models for FAIR as well as selective and non-selective DIR-labeling are provided in Appendix A (Equations (A1)-(A3), (A8)-(A9), and (A11)-(A12), respectively). Subsequently, the MBF was calculated based on Buxton's GKM [2, 3]. The correlation between the obtained MBF, PN, and SNR values and the HR variability ($\sigma_{RR}$) was evaluated using Spearman's correlation. Slope and intercept values were obtained from a linear regression of phantom values and are reported with a 95 % confidence interval (CI).

# Numerical Simulations: Results

A summary of the coefficient of determination ($R^2 = \rho^2_{Spearman}$) and the linear regression results of the myocardial blood flow (MBF), physiological noise (PN), and SNR gain can be found in Supporting Information Tables S1, S2, and S3, respectively. Regression slope and intercept values are reported with the corresponding 95 % confidence intervals and $p$-values. Supporting Information Figure S1 shows the MBF values obtained from simulation experiments with conventional FAIR and DIR-myoASL. Across the range of simulated HR variability, $\sigma_{RR}$, and inversion efficiency, the simulated MBF values remained largely constant at 2.5 ml/g/min for single FAIR ($0.0 < R^2 < 0.03$, -0.07 [-0.11, -0.01] < slope [CI] < 0.13 [-0.03, 0.05], $0.01 < p_{slope} < 0.67$, $p_{intercept} < 0.001$) and selective DIR-preparations ($0.04 < R^2 < 0.34$, -0.03 [-0.04, -0.02] < slope [CI] < -0.01 [-0.02, 0.01], $0.001 < p_{slope} < 0.37$, $p_{intercept} < 0.001$). Non-selective DIR-labeling resulted in slightly lower perfusion values, around 2.0 ml/g/min ($R^2$=0.25, CI: [1.94, 1.99], $p_{intercept} < 0.001$) at 85 % inversion efficiency, but remained almost constant across the all simulated HR variabilities ($0.16 < R^2 < 0.53$, -0.03 [-0.05, -0.01] < slope [CI] < -0.01 [-0.02, 0.00], $0.001 < p_{slope} < 0.03$). The statistical comparison showed significant differences in MBF values between non-selective DIR and both conventional FAIR ($p < 0.001$) and selective DIR ($p < 0.001$). However, these differences were reduced with improved inversion efficiency and were fully eliminated for perfect inversion.

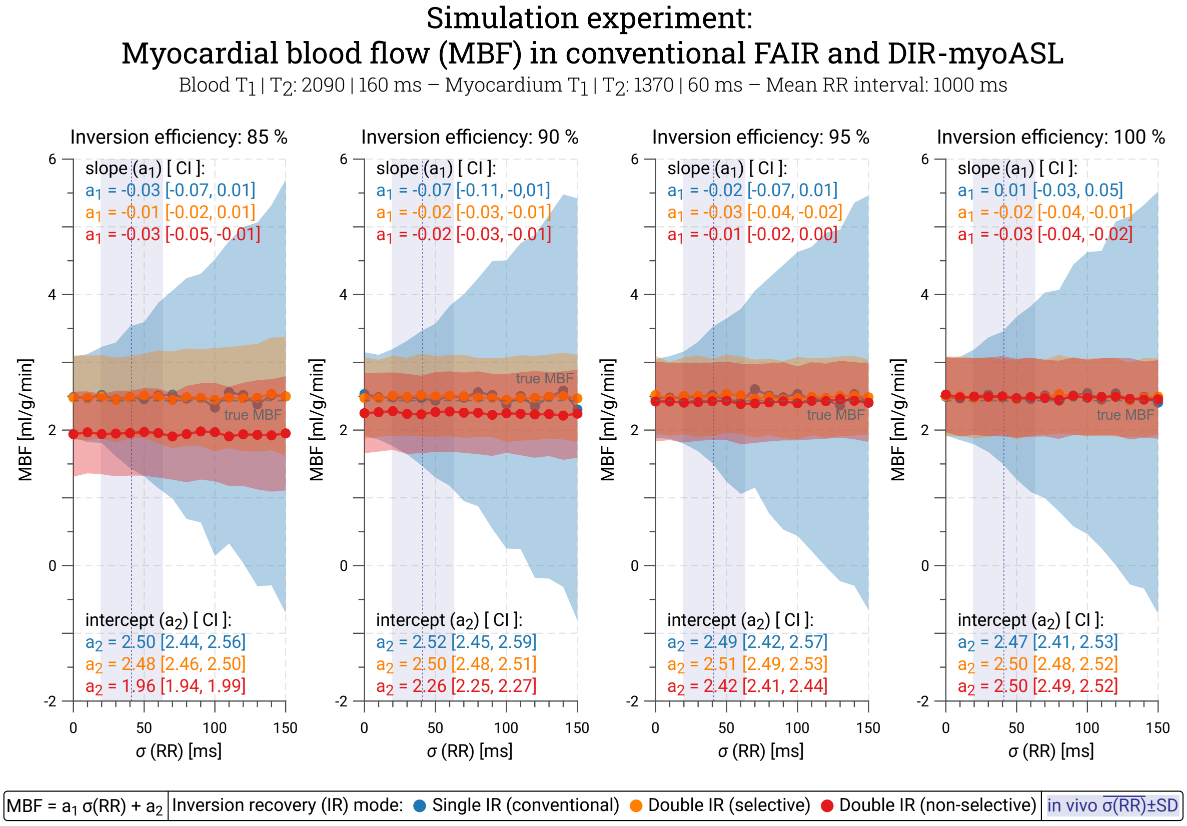


**Figure S1:** Simulated myocardial blood flow (MBF) obtained with double inversion recovery (DIR; orange, red) and a conventional FAIR-myoASL sequence (blue). The MBF is shown as a function of the simulated heart rate variability $\sigma_{RR}$. The linear regression slope ($a_1$, in units of 10 s$^{-1}$ ) and intercept ($a_2$) are reported with the corresponding 95 % confidence interval (CI). The average HR variability ±SD as observed in our in vivo study is highlighted in purple. The MBF is largely constant over the range of simulated $\sigma_{RR}$ ($0.02 < R^2 < 0.53$, slope: -0.07-0.01 across all sequences), showing larger fluctuations for conventional compared to DIR-labeling. Non-selective reinversion pulses lead to decreasing perfusion values for lower inversion efficiencies (intercept: 1.96-2.50) compared to conventional FAIR-labeling and selective reinversion (intercept: 2.47-2.50).

The effect of a variable heart rate on the PN in simulations is illustrated in Supporting Information Figure S2. The PN is determined as the standard deviation of simulated perfusion values and is shown as a function of $\sigma_{RR}$. The slope as obtained from linear regression analysis is given in units of $10s^{-1}$. For conventional FAIR-myoASL, the PN increased with increasing $\sigma_{RR}$ across all levels of inversion efficiency ($R^2$=1.0, 1.82 [1.77, 1.87] < slope [CI] < 1.84 [1.78, 1.89], $p_{slope}$ < 0.001). However, for selective and non-selective DIR-labeling, the increase of PN with $\sigma_{RR}$ progressively decreased as inversion efficiency increased. At 85 % inversion efficiency, the regression slope was 0.24 ($R^2$=1.0, CI: [0.22, 0.26], $p_{slope}$ < 0.001) with both selective and non-selective reinversion pulses. Whereas at 100 % inversion efficiency, PN remained largely constant for both types of reinversion pulses ($R^2$=0.58/$R^2$=0.42 selective/non-selective DIR, slope: 0.01, CI: [0.00, 0.01], $p_{slope}$< 0.001). Overall, PN values were nearly identical when comparing selective and non-selective DIR-preparations.

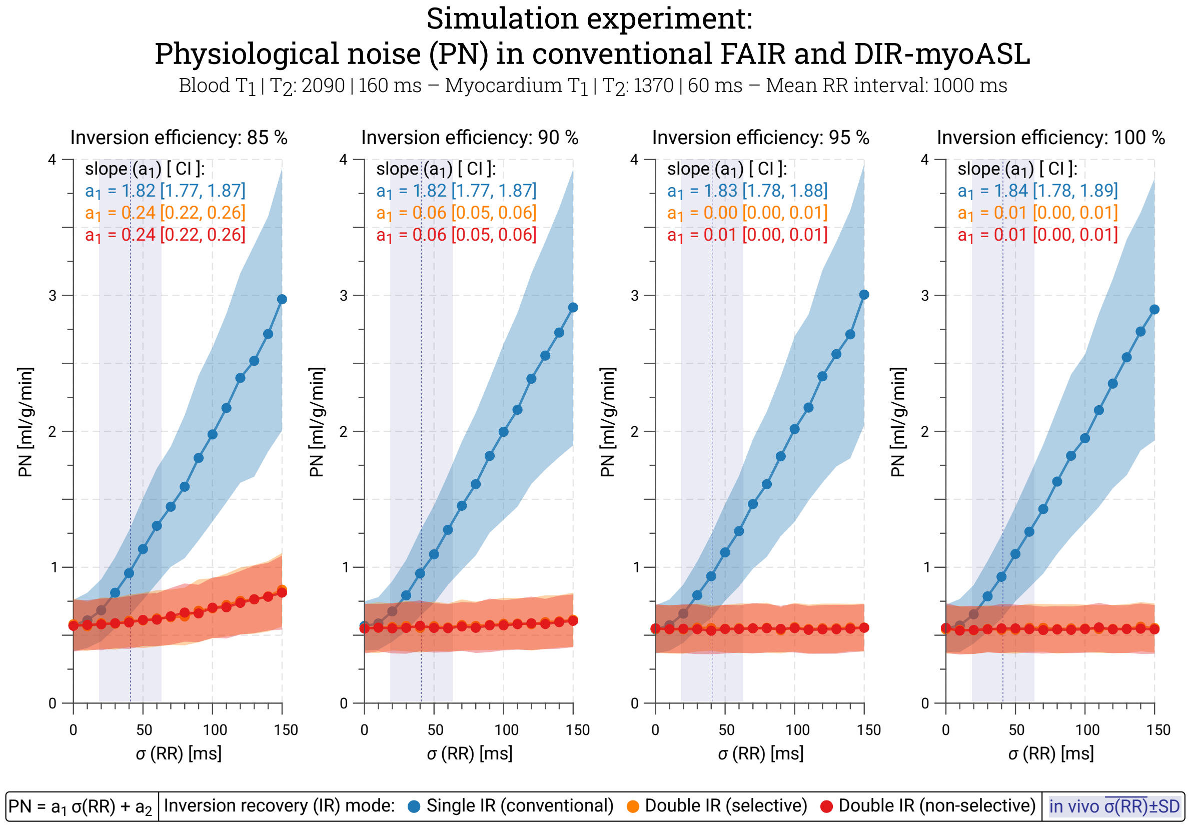


**Figure S2:** Physiological noise (PN) determined as the standard deviation of simulated perfusion values obtained with double inversion recovery (DIR; orange, red) and a conventional FAIR-myoASL sequence (blue). PN is shown as a function of the simulated heart rate variability $\sigma_{RR}$. The linear regression slope ($a_1$) is reported with the corresponding 95 % confidence interval (CI) in units of 10 $s^{-1}$. The average HR variability ±SD as observed in our in vivo study is highlighted in purple. With DIR-labeling the PN increases slightly with increasing $\sigma_{RR}$ (0.07 < $R^2$ < 1.0, slope: 0.01-0.24 for both types of DIR-labeling) at low inversion efficiencies, whereas for conventional myoASL PN shows a much stronger increase with $\sigma_{RR}$ ($R^2$=1.0, slope: 1.82-1.84).

| Inversion efficiency | | **FAIR** | **selective DIR** | **non-selective DIR** |
|---|---|---|---|---|
| 85 % | $R^2$: | 0.03 | 0.99 | 0.97 |
| | slope: | -0.03 [-0.07, -0.01], $p$=0.18 | -0.02 [-0.02, -0.01], $p$=0.37 | -0.03 [-0.05, -0.01], $p$ <0.05 |
| | intercept: | 2.50 [2.44, 2.56], $p$ <0.05 | 2.48 [2.46, 2.50], $p$ <0.05 | 1.96 [1.94, 1.99], $p$ <0.05 |
| 90 % | $R^2$: | 0.15 | 0.97 | 0.99 |
| | slope: | -0.06 [-0.11, -0.01], $p$ <0.05 | -0.02 [-0.03, -0.01], $p$ <0.05 | -0.03 [-0.03, -0.01], $p$ <0.05 |
| | intercept: | 2.52 [2.45, 2.59], $p$ <0.05 | 2.50 [2.48, 2.51], $p$ <0.05 | 2.26 [2.25, 2.27], $p$ <0.05 |
| 95 % | $R^2$: | 0.02 | 0.06 | 0.99 |
| | slope: | -0.02 [-0.07, 0.03], $p$=0.46 | -0.03 [-0.04, -0.02], $p$ <0.05 | -0.02 [-0.02, -0.01], $p$ <0.05 |
| | intercept: | 2.49 [2.42, 2.57], $p$ <0.05 | 2.50 [2.49, 2.51], $p$ <0.05 | 2.42 [2.41, 2.44], $p$ <0.05 |
| 100 % | $R^2$: | 0.00 | 0.58 | 0.99 |
| | slope: | 0.01 [-0.03, 0.05], $p$=0.69 | -0.04 [-0.05, -0.04], $p$ <0.05 | -0.04 [-0.04, -0.02], $p$ <0.05 |
| | intercept: | 2.47 [2.41, 2.53], $p$ <0.05 | 2.50 [2.48, 2.52], $p$ <0.05 | 2.50 [2.49, 2.52], $p$ <0.05 |

**Table S1:** Slope and intercept values as obtained from linear regression of simulated MBF and RR variability. Values are reported with the corresponding coefficient of determination ($R^2$), p-values, and 95 % confidence intervals (CI). The linear regression slope is given in units of 10 $s^{-1}$.

| Inversion efficiency | | **FAIR** | **Selective DIR** | **Non-Selective DIR** |
|---|---|---|---|---|
| 85 % | $R^2$: | 1.00 | 1.00 | 1.00 |
| | slope: | 1.82 [1.77, 1.87], $p < 0.05$ | 0.24 [0.22, 0.25], $p < 0.05$ | 0.24 [0.22, 0.25], $p < 0.05$ |
| | intercept: | 0.26 [0.18, 0.34], $p < 0.05$ | 0.49 [0.46, 0.52], $p < 0.05$ | 0.49 [0.46, 0.52], $p < 0.05$ |
| 90 % | $R^2$: | 1.00 | 0.97 | 0.95 |
| | slope: | 1.82 [1.77, 1.87], $p < 0.05$ | 0.06 [0.05, 0.06], $p < 0.05$ | 0.06 [0.05, 0.06], $p < 0.05$ |
| | intercept: | 0.26 [0.18, 0.33], $p < 0.05$ | 0.54 [0.52, 0.54], $p < 0.05$ | 0.53 [0.52, 0.54], $p < 0.05$ |
| 95 % | $R^2$: | 1.00 | 0.06 | 0.54 |
| | slope: | 1.82 [1.77, 1.88], $p < 0.05$ | 0.00 [0.00, 0.01], $p < 0.05$ | 0.01 [0.00, 0.01], $p < 0.05$ |
| | intercept: | 0.25 [0.18, 0.32], $p < 0.05$ | 0.55 [0.54, 0.55], $p < 0.05$ | 0.54 [0.54, 0.55], $p < 0.05$ |
| 100 % | $R^2$: | 1.00 | 0.58 | 0.42 |
| | slope: | 1.84 [1.78, 1.89], $p < 0.05$ | 0.01 [0.00, 0.01], $p < 0.05$ | 0.01 [0.00, 0.01], $p < 0.05$ |
| | intercept: | 0.23 [0.15, 0.31], $p < 0.05$ | 0.54 [0.54, 0.54], $p < 0.05$ | 0.54 [0.54, 0.54], $p < 0.05$ |

**Table S2:** Slope and intercept values as obtained from linear regression of simulated PN and RR variability. Values are reported with the corresponding coefficient of determination ($R^2$), p-values, and 95 % confidence intervals (CI). The linear regression slope is given in units of 10 $s^{-1}$.

| Inversion efficiency | | **FAIR** | **Selective DIR** | **Non-Selective DIR** |
|---|---|---|---|---|
| 85 % | $R^2$: | 0.05 | 0.98 | 0.97 |
| | slope: | 0.00 [0.00, 0.00], $p$=1 | 1.44 [1.31, 1.57], $p$ <0.05 | 1.12 [1.01, 1.22], $p$ <0.05 |
| | intercept: | 1.00 [1.00, 1.00], $p$ <0.05 | 1.18 [0.99, 1.37], $p$ <0.05 | 0.94 [0.78, 1.10], $p$ <0.05 |
| 90 % | $R^2$: | 0.02 | 0.99 | 0.99 |
| | slope: | 0.00 [0.00, 0.00], $p$=1 | 2.71 [2.59, 2.82], $p$ <0.05 | 2.44 [2.33, 2.55], $p$ <0.05 |
| | intercept: | 1.00 [1.00, 1.00], $p$ <0.05 | 0.72 [0.55, 0.89], $p$ <0.05 | 0.70 [0.54, 0.86], $p$ <0.05 |
| 95 % | $R^2$: | 0.01 | 0.99 | 0.99 |
| | slope: | 0.00 [0.00, 0.00], $p$=1 | 3.30 [3.09, 3.51], $p$=0.15 | 3.18 [3.01, 3.36], $p$ <0.05 |
| | intercept: | 1.00 [1.00, 1.00], $p$ <0.05 | 0.47 [0.17, 0.78], $p$ <0.05 | 0.49 [0.23, 0.75], $p$ <0.05 |
| 100 % | $R^2$: | 0.03 | 1.00 | 0.99 |
| | slope: | 0.00 [0.00, 0.00], $p$=1 | 3.12 [2.99, 3.25], $p$ <0.05 | 3.14 [3.00, 3.28], $p$ <0.05 |
| | intercept: | 1.00 [1.00, 1.00], $p$ <0.05 | 0.59 [0.40, 0.78], $p$ <0.05 | 0.58 [0.38, 0.79], $p$ <0.05 |

**Table S3:** Slope and intercept values as obtained from linear regression of simulated SNR gain and RR variability. Values are reported with the corresponding coefficient of determination ($R^2$), p-values, and 95 % confidence intervals (CI). The linear regression slope is given in units of 10 $s^{-1}$.

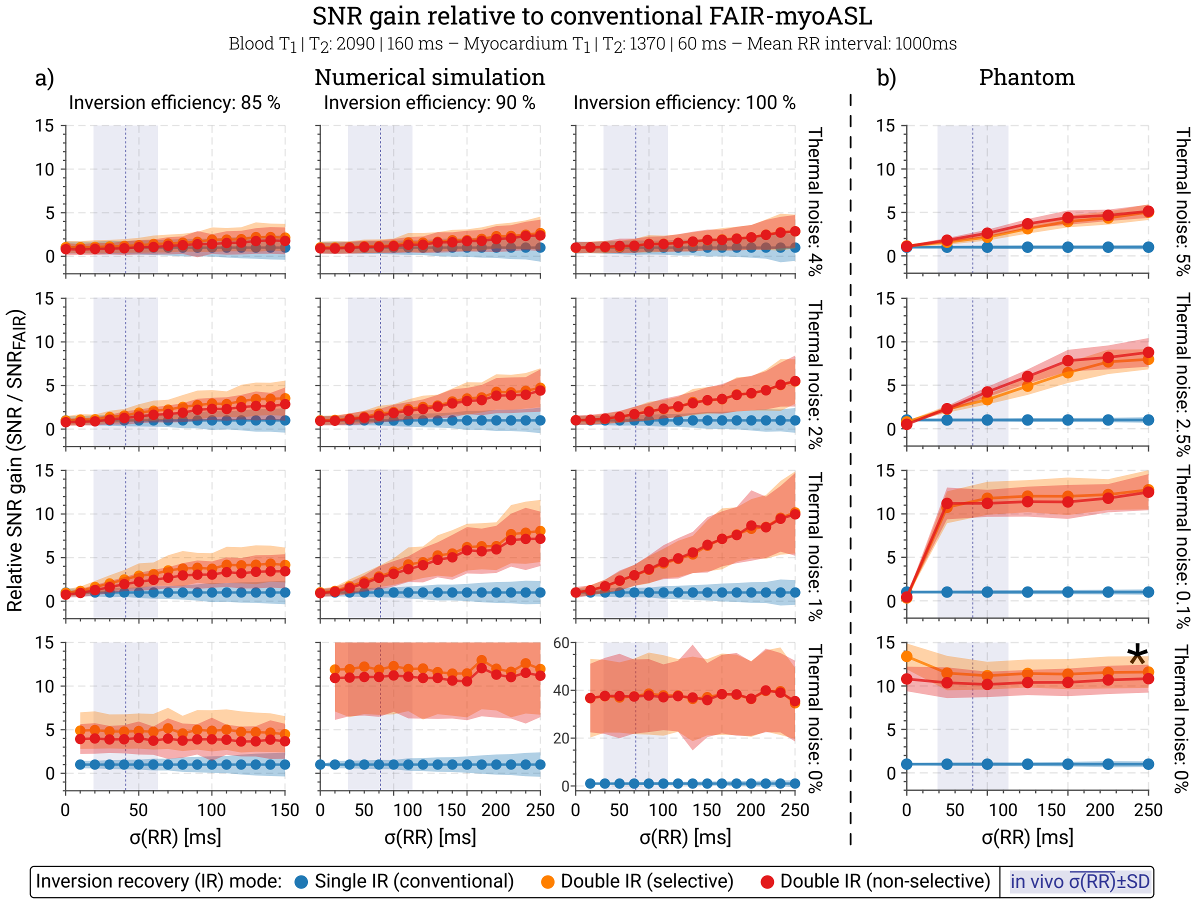


**Figure S3:** (a) Simulated and (b) phantom SNR gain relative to conventional FAIR-myoASL as a function of RR interval variability ($\sigma(RR)$), mimicking physiological noise. The range of RR variabilities as observed in our in vivo study (mean±SD: 44±22 ms) is highlighted in purple. The SNR gain is shown across different levels of thermal noise and, for simulations, across varying inversion efficiency. Thermal noise levels were defined as the ratio of the (background) standard deviation to the intensity of the baseline signal. In phantom, thermal noise was eliminated by deriving all signals from the same images (indicated with asterisk). In both phantom and simulations, the SNR gain achieved with DIR-labeling increased with increasing ratio of physiological to thermal noise. In the absence of thermal noise, the SNR gain remained constant across the range of simulated RR variability and increased for higher inversion efficiency.

# Phantom experiments: Results

The effect of a variable HR on myoASL-measurements using conventional FAIR and DIR-labeling in phantom experiments is illustrated in Supporting Information Figure S4. Across the range of simulated HR variabilities ($\sigma_{RR}$), the average MBF values remained largely constant at around 3.3 ml/g/min for all three myoASL sequences ($0.41 < R^2 < 1.0$, -0.16 [-0.25, -0.07] < slope [CI] < 0.03 [0.01, 0.04], $p <$ 0.05). The PN increased with higher HR variability. At a variability level corresponding to the mean $\sigma_{RR}$ observed in the in vivo results, conventional FAIR-labeling yielded a mean PN 0.46±0.08 ml/g/min ($R^2$=1.0, slope [CI]: 0.74 [0.63, 0.84], $p < 0.001$). At the same HR variability level, both DIR-labeling methods showed lower mean PN (0.14±0.02 ml/g/min), as well as an overall reduced dependence on $\sigma_{RR}$ ($R^2$=1.0, slope [CI]: 0.04 [0.03, 0.06], $p < 0.001$). Compared to conventional FAIR-labeling, substantial gains in SNR were observed for both DIR-labeling methods. The SNR gain increased with the HR variability when using both selective ($R^2$=0.98, slope [CI]: 3.49 [2.70, 4.28], $p$< 0.001) and non-selective reinversion pulses ($R^2$=1.0, slope [CI]: 3.69 [2.64, 4.74], $p < 0.001$). For a $\sigma_{RR}$ corresponding to the mean HR variability in vivo, this led to a relative SNR gain of 2.91±0.52 for selective and 3.56±0.53 for non-selective DIR labeling. Similar to the simulation results, the SNR gain in phantom remained constant in the absence of thermal noise and decreased with increasing thermal noise levels across the range of $\sigma_{RR}$ (Supporting Information Figure S3).

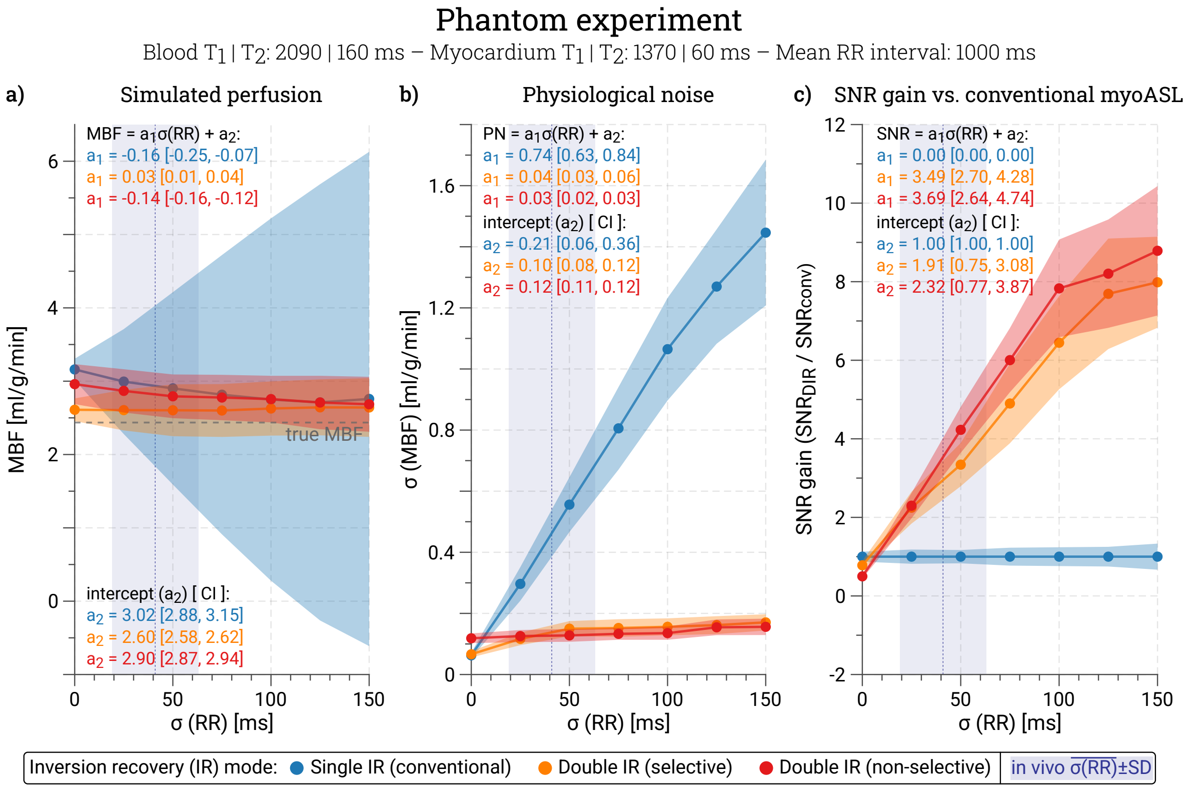


**Figure S4:** (a) Myocardial blood flow (MBF), (b) physiological noise (PN), and (c) relative SNR gain obtained from phantom experiments. Data is shown as a function of the heart rate (HR) variability for conventional (blue), selective (orange), and non-selective (red) double inversion labeling. The linear regression slopes ($a_1$, in units of 10 s$^{-1}$) and intercepts ($a_2$) are reported with the corresponding 95 % confidence interval (CI). The average HR variability ±SD as observed in our in vivo study is highlighted in purple. PN was induced by combining control and tag images acquired at different HR. PN was then calculated as the standard deviation across control-tag pairs. Lower PN and higher SNR were achieved for double inversion preparations compared with conventional labeling, while the MBF values remained comparable.

# Correlation of PN with RR variability in vivo

The results of the correlation and linear regression analysis of in vivo PN versus RR variability are summarized in Supporting Information Tables S4 and S5, respectively. For each labeling strategy, Spearman's correlation coefficients ($\rho_{Spearman}$) are reported with the corresponding p-values. Linear regression slopes and intercepts are given with the respective p-values and 95 % confidence intervals (CI).

| | **FAIR** | **selective DIR** | **non-selective DIR** |
|---|---|---|---|
| $\rho_{Spearman}$ | 0.75 | 0.59 | 0.66 |
| $p_{Spearman}$ | <0.05 | <0.05 | <0.05 |

**Table S4:** Spearman's correlation coefficients ($\rho_{Spearman}$) and p-values between in vivo physiological noise (PN) and variability in RR duration for the investigated labeling strategies. FAIR-labeling showed a statistically significant, strong positive correlation, while DIR-labeling showed significant but more moderate correlations.

| | **FAIR** | **selective DIR** | **non-selective DIR** |
|---|---|---|---|
| Slope | 2.30 | 0.70 | 0.88 |
| $p_{slope}$ | <0.05 | <0.05 | <0.05 |
| $CI_{slope}$ | [1.48, 3.12] | [0.33, 1.07] | [0.52, 1.24] |
| Intercept | -0.04 | 0.16 | 0.20 |
| $p_{intercept}$ | 0.83 | 0.05 | <0.05 |
| $CI_{intercept}$ | [-0.42, 0.34] | [0.00, 0.31] | [0.02, 0.37] |

**Table S5:** Linear regression slope and intercept values of in vivo PN versus RR variability, with the corresponding p-values and 95 % confidence intervals (CI). The linear regression slope is reported in units of 10 $s^{-1}$. FAIR-labeling exhibited an approximately 3.3-fold/2.5-fold higher slope compared to selective/non-selective DIR-labeling.

# References


[1] J. Z. Bojorquez et al. “What are normal relaxation times of tissues at 3 T?” In: Magnetic resonance imaging 35 (2017), pp. 69–80.

[2] M. Božić-Iven et al. “Improved reproducibility for myocardial ASL: Impact of physiological and acquisition parameters”. In: Magnetic Resonance in Medicine 91.1 (2024), pp. 118–132.

[3] R. B. Buxton et al. “A general kinetic model for quantitative perfusion imaging with arterial spin labeling”. In: Magnetic Resonance in Medicine 40.3 (1998), pp. 383–396.

[4] G. J. Stanisz et al. “T1, T2 relaxation and magnetization transfer in tissue at 3T”. In: Magnetic Resonance in Medicine 54.3 (2005), pp. 507–512.

[5] S. Weingärtner et al. “Myocardial T1-mapping at 3T using saturation-recovery: reference values, precision and comparison with MOLLI”. In: Journal of Cardiovascular Magnetic Resonance 18.1 (2017), pp. 1–9.
